# Perceived Versus Real Swimming Skills of Adolescents under Standard and Challenging Conditions: Exploring Water Competencies as an Approach to Drowning Prevention

**DOI:** 10.3390/ijerph17113826

**Published:** 2020-05-28

**Authors:** Marek Rejman, Anna Kwaśna, Magdalena Chrobot, Per-Ludvik Kjendlie, Robert K Stalmann

**Affiliations:** 1 Department of Physical Education, University School of Physical Education in Wroclaw, 51-612 Wroclaw, Poland; anna.kwasna@awf.wroc.pl (A.K.); magdalena.chrobot@awf.wroc.pl (M.C.); 2 Department of Physical Performance, Norwegian Police University College, 0369 Oslo, Norway; per-ludvik.kjendlie@phs.no; 3 Department of Physical Performance, Norwegian School of Sport Sciences, 0863 Oslo, Norway; robertkeig.stallman@gmail.com

**Keywords:** swimming, drowning prevention, adolescents, self-assessment, aquatic education

## Abstract

In this study, we compared adolescents’ actual (expert assessed) front crawl swimming skills to their self-assessment in two conditions: in standard swimming (wearing a swimsuit and goggles) and in a simulated risk scenario (swimming in plain clothes without goggles). We postulated that education focused on water competencies is fundamental in preventing drownings. Experts evaluated the skills of 21 female and 21 male adolescents in both standard and challenging conditions. All were low-skilled swimmers aged 14–15 years. Participants were asked to self-assess their skills before and after each trial. Boys and girls covered the same distance in both trials. Their self-assessment did not change regardless of the difficulty of the conditions. Girls assessed themselves more accurately than boys. However, boys who underestimated their skills showed greater ability to utilise the experience gained from performing the task for a more accurate self-assessment. In conclusion, adolescents should be educated in total water competencies, and not merely in swimming skills. For girls, “water readiness” is thought to broaden their ability to adapt their swimming skills to nonstandard conditions. Aquatic education for boys should focus on developing self-reflection in order to create a long-lasting responsibility using their own swimming skills.

## 1. Introduction

According to the World Health Organization (WHO) Drowning Report [1], approximately 360,000 people drown each year. Of these individuals, over half are under 25 years of age. This worrying statistic highlights the importance of drowning prevention education, especially among children and adolescents. We believe that swimming education is one of the most important interventions in reversing this fatal tendency. Among the youth whose lives were lost in water, approximately 10% lived in high-income countries where the opportunity to learn to swim is common, either at school or through various organizations that offer swimming classes. Therefore, our question is: why do so many people drown in these countries? Phrasing the question like this places the emphasis on learning to swim safely as an essential part of aquatic education and as an important drowning prevention intervention.

### 1.1. Water Safety Education

Water safety education is aimed at adopting safe behaviours and attitudes in, on, and around the water beginning from an early age as well as to maintain such attributes throughout life. This education involves the formation of positive habits related to the responsibilities and consequences of aquatic activities [2,3]. In order to have an educational impact, aquatic activities should be integrated into the educational curriculum, and must aim to evoke permanent changes in values, attitudes, knowledge, judgment, and behaviours. Within these aquatic activities, affective, cognitive, and psychomotor competencies must be creatively integrated with one another [4]. 

According to Newell’s [5] theory of dynamic constraints in motor learning [6,7], water safety education is a dynamic process in which the effectiveness is the result of a multilevel interaction between the individual, the environment, and the task at hand [8]. Furthermore, water safety education is aligned (associated) with the ecological model of healthy behaviour [9], while a certain pedagogical optimism, supported by experiments, confirms that young people may be influenced in this manner [2]. 

In the USA, drowning is the number one cause of unintentional injury-related death of children and adolescents up to 19 years of age [10]. For this reason, the focus of this study was on teenaged youth. The causes of drowning listed by the WHO [1] unequivocally fit into the negative aquatic attitudes and behaviours of this age group [3]. Thus, poor swimming, self-rescue, and lifesaving skills, together with a low level of awareness of danger that can progress to life-threatening situations, have become the main focuses of water safety education.

### 1.2. Self-Assessment, Water Safety Awareness, and Water Competencies 

Efficient propulsion, measured by the distance covered, among other means, is the key to extending survival time if one must swim under high-risk conditions [4,8]. Thus, efficient propulsion, not in the context of competitive swimming, is an essential aspect of water safety [4,8,11,12]. A number of authors have reported a decline in efficiency when changing from standard swimming conditions (i.e., swimming pool) to other challenging situations such as moving from warm water to cold water [13,14], from calm water to waves [15], or when wearing swimwear versus plain clothing [16,17,18,19]. This provides a partial answer to why “good swimmers” often fail to save themselves in unfamiliar swimming conditions (i.e., not in a pool). In this study, the ability to adapt the swimming skills to environmental constraints was considered in the context of swimming while wearing plain clothes versus swimming gear. 

It is well known (17) that the increase in the water resistance and the reduction in the mobility of the propulsive body parts are the main factors determining the effect of clothing on swimming performance. Choi et al. [18], Ohkuwa et al. [19], and Moran [16] demonstrated a significant increase in the energetic and psychological cost and decrease in distance covered when swimming in clothes. The first attempt to investigate the link between various swimming strokes performed under standard conditions and the ability to cope with various task and environmental constraints (swimming in clothes) among novice swimmers was made by Potdevin et al. [20]. The authors showed that the difference between clothed vs. unclothed swimming is stroke dependent. The breaststroke was less affected and the front crawl was the most affected by wearing clothes in performance on the maximal distance. The backstroke was allowed to maintain a horizontal position for the longest period of time.

Moran et al. [11] concluded that one of the main causes of drowning among young people is overestimation of their own self-assessed swimming skills, together with underestimation of the risk. A growing number of studies have confirmed that this is also the case for children, adolescents, and even parents and teachers who inaccurately judge the skills of their own children and pupils [21,22,23]. Given this recent evidence, the ability to accurately assess one’s own swimming skills has been identified as an essential part of water safety, i.e., one that offers protective value and reduces risk [4].

According to cognitive and constructivist theories of learning and motivation, self-assessment is defined as the ability to realistically evaluate one’s own skills [24]. Self-assessment measures the individual’s perceptions of their own abilities and accomplishments [25] and is correlated with the general attitude of students toward education [26,27] including physical activity [28]. Self-assessment of motor skills can be of crucial importance for self-esteem and has also been found to be associated with the behaviour of the individual [29,30]. It is, therefore, possible that a strong positive correlation between students’ perception of their own swimming skills and their real swimming skills may result not only in higher levels of physical fitness and participation in aquatic activities, but also in positive attitudes and behaviours in an aquatic setting. Therefore, in this study, the issue of education on safety in and around water will be considered through the prism of water competencies acquired by youth as part of their school curriculum. 

Water competencies involve the individual’s cognitive, affective, and psychomotor experiences [31] in relation to water environments [4], which enables them to effectively apply the necessary skills under varying conditions, including challenges caused by internal factors (e.g., emotions and fatigue) and external factors (e.g., specific swimming conditions like temperature, clothing, waves, current, wind, etc.) in order to reduce the risk of drowning. It includes keeping both yourself and others safe [4,11,32]. Water competencies evolve by attaining knowledge and behavioural experiences, which involves a certain proficiency in a set of skills comprising a system of values, attitudes, and behaviours. These skills promote rational behaviour in and around water. In contrast to swimming skills alone, it is not enough to simply “learn” them [12]. Although they represent an important part of water competencies, skills alone are often not sufficient to prevent drowning [10]. Consequently, an educational intervention based solely on learning physical swimming skills might actually impair the learner’s ability to act safely in an aquatic environment, as previously suggested [10,11,12]. 

In the current study, we evaluated swimming skills among adolescents (males and females) under standard swimming conditions (performed wearing a swimsuit and goggles) and under a challenge scenario (swimming in clothes without goggles). The assessment was based on a subjective self-assessment of the participants’ own perceived beliefs about their swimming skills, which was compared to their “real skills,” as assessed based on objective criteria by experts. A unique aspect of this study is that the self-assessment of perceived swimming skills was conducted twice, before and after each trial, when the participants had gained practical experience and information related to the trial itself. 

The aim of the study was to perform an unbiased assessment of front crawl swimming skills of adolescents based on the distance covered under standard conditions (wearing a swimsuit and goggles) and in a simulated risk scenario (swimming in plain clothes without goggles). A comparison of adolescents’ real swimming skills with their self-assessment was also performed for both situations. Assuming that a lack of ability to objectively predict the skills level could lead to life-threatening situations, we postulated that education focused on water competencies is fundamental in preventing drownings. 

The following research questions were formulated and research tasks were established (with gender differences considered). Numbered lists can be added as follows.

1. To investigate the real protective value of swimming skills in adolescents, we compared the maximal distance covered in two trials (swimming under standard conditions (wearing swimwear and goggles) and under a more challenging situation (wearing clothes without goggles)).
2. Analysis of self-assessment scores was performed to reveal how adolescents perceive their own swimming skills under standard conditions (wearing swimwear and goggles) and in a potential risk scenario simulation (swimming in clothes without goggles).
3. Adolescents’ self-assessment scores before and after the experimental trials were compared to determine whether they are able to learn from experience gained from practical implementation of the task to more accurately assess their swimming skill.
4. The differences between adolescents’ self-perceived skills and their real skills (assessed by experts) were evaluated to determine whether they can accurately assess their own swimming skills under standard and challenging conditions. Taking this into consideration, the awareness of swimming skills – a crucial part of water competencies - could be diagnosed.
5. The relationship between adolescents’ self-assessment and their actual swimming skills (assessed by experts) was studied to reveal whether the real level of swimming skills goes hand-in-hand with the accuracy of self-perception of these skills.

Having the answers to these questions should tell us more about water competencies of adolescents in terms of knowledge, behaviour, and attitude toward water safety. 

## 2. Materials and Methods

### 2.1. Participants

The participants included 21 boys and 21 girls aged 14–15 years recruited from the three schools of the same district in a city of 800,000 inhabitants. All of them voluntary participated as one group in a basic swimming course (thirteen 45-min lessons held once a week). The learning program led by the same two swimming teachers included the first stage called “water readiness,” as well as backstroke and front crawl swimming. It was assumed that all adolescents participating in the course had similar levels of aquatic experience and knowledge, which was reflected in their individual swimming skills. Although the participants differed from one another, this was not considered to be an issue because each subject was controlling and assessing their own swimming skills in the repeated trials. Therefore, the water experience accumulated by each adolescent before the research session was not assessed. All the participants were healthy, and no one reported any temporary indisposition. Therefore, it seems that their level of fitness on the day of the test was optimal.

### 2.2. Experimental Design and Tasks

In the first trial, in a random order, participants swam as far as possible (up to 25 m) using front crawl swimming stroke while wearing a typical swimming suit and goggles. After a 30-min rest, an identical procedure was conducted with the same participants, but they were asked to swim in clothes (sweatpants and sweatshirt with long sleeves) without goggles. The time interval between individual trials eliminated the impact of fatigue on the quality of their performance. 

Prior to the first trial, participants were asked about any previous experience in swimming in clothes. They all replied negatively. Swimming in clothes was considered a “novel” task. All trials were performed in a 25-m indoor swimming pool (depth 1.5 to 1.8 m) with the water temperature maintained at 27 ± 1 °C. Evaluation of the level of swimming skills of adolescents was based solely on the distance covered (up to 25 m with an accuracy of 1 m). Performance was evaluated objectively using the Delphi method, modified by Keegan et al., with the participation of three experts [33,34].

The distance covered by each student separately was a main criterion of the assessment. The end of the trial was defined when the students stopped swimming (stood on the bottom and grabbed the rope or the edge of the pool). Additionally, the experts paid attention to cyclical coordination of the arm stroke. Keeping the elbows above water with no “dog paddle” was also required. The participants’ breathing control and head position were not taken into consideration. They swam using the front crawl stroke at a self-determined pace. The distance covered, assessed in this manner, was considered a measure of the level of swimming skills in the current study [4,8,11,12]. The tasks were intentionally arranged to give the adolescents an opportunity to perform both trials in the simplest, most natural way, which provided optimal conditions for assessing their real swimming skills level.

In both parts of the study, participants were asked to self-assess their performance before and after the trial. This self-assessment was based on each respondent’s answer to the question: “how do you assess your swimming skills on a point scale from 0 to 5?” The question was intentionally formulated in this simple way to focus the adolescents on “blind” (natural) self-assessment, which was independent from the reliable assessment criteria taken into consideration by the experts. The self-assessment interview was performed with each respondent separately and face-to-face without third parties by an independent assistant. It was assumed that this procedure ensured the reliability of self-assessment. The self-assessment scale (from 0 to 5) corresponded to the assessment scale for swimming skills. In this way, we could compare real and perceived performance in swimwear and in clothes. A summary of the trials and the assessment scale for the results are presented in Table 1.

All study procedures involving human participants were performed in accordance with the ethical standards of the institutional research committee (Ethical Committee of the University School of Physical Education in Wroclaw, Poland, reference number 126/2018) and with the 1964 Declaration of Helsinki and its later amendments or comparable ethical standards. The juvenile participants and their parents or caregivers were informed about the objectives and procedures of the experiment. Parental informed consent for participation in the study was obtained.

### 2.3. Statistical Analysis

Statistical analyses were performed using Statistica 13.0 software (StatSoft, Tulsa, OK, USA). All variables fulfilled the prerequisites of the parametric test for normally distributed data, which was assessed using the Shapiro-Wilk test. The homogeneity of variance was also confirmed by Levene’s variance ratio test. Paired *t*-tests, performed separately for the group of girls (*n* = 21) and boys (*n* = 21), were used to compare the differences in assessment of front crawl swimming skills performed in swimwear and in clothes without goggles. The same statistical tool was used to compare the differences in self-assessment of these adolescents before and after the previously mentioned trials. One-way ANOVA (based on gender) followed by the Newman-Keuls post hoc test was used for intergroup analysis. Pearson’s correlation coefficients were computed to assess the level of association (*p* ≤ 0.05) between pairs of variables. Correlation effect sizes were deemed as: weak, 0 < |R| ≤ 0.1, small, 0.1 < |R| ≤ 0.3, moderate, 0.3 < |R| ≤ 0.5, or strong, |R| > 0.5.

## 3. Results

**Figure 1:**
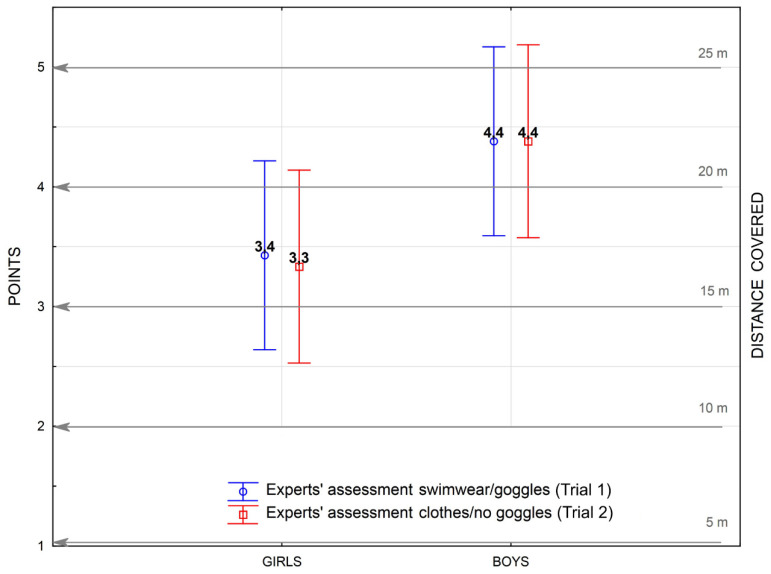
Comparison of mean reliable (expert) assessment scores of the swimming skills of girls and boys in crawl swimming trials under standard (swimwear and goggles) and challenging (wearing clothes without goggles) conditions in terms of the distance covered.

According to the objective assessment of swimming skills by experts (Figure 1), girls swam a distance of about 17 m (16.75 ± 3.65 m) regardless of whether they were wearing swimwear or plain clothes (achieving 3.3–3.4 (±0.73) points in both trials). For both the standard swimwear and plain clothes trials, boys swam a longer distance than girls (22.0 ± 4.1 m) and obtained an average score of 4.4 (±0.82) points in both trials. However, the difference (5 m) between males and females was not statistically significant (Table 2). There were also no differences in the scores obtained for girls and boys when assessed separately (Table 2).

There were no statistically significant differences in the self-assessment scores of adolescents’ own swimming skills under standard (swimwear and goggles) and challenging (clothes and no goggles) conditions (Table 3). In addition, there were also no differences in self-assessment scores before and after both of the previously mentioned trials (Table 3).

**Figure 2:**
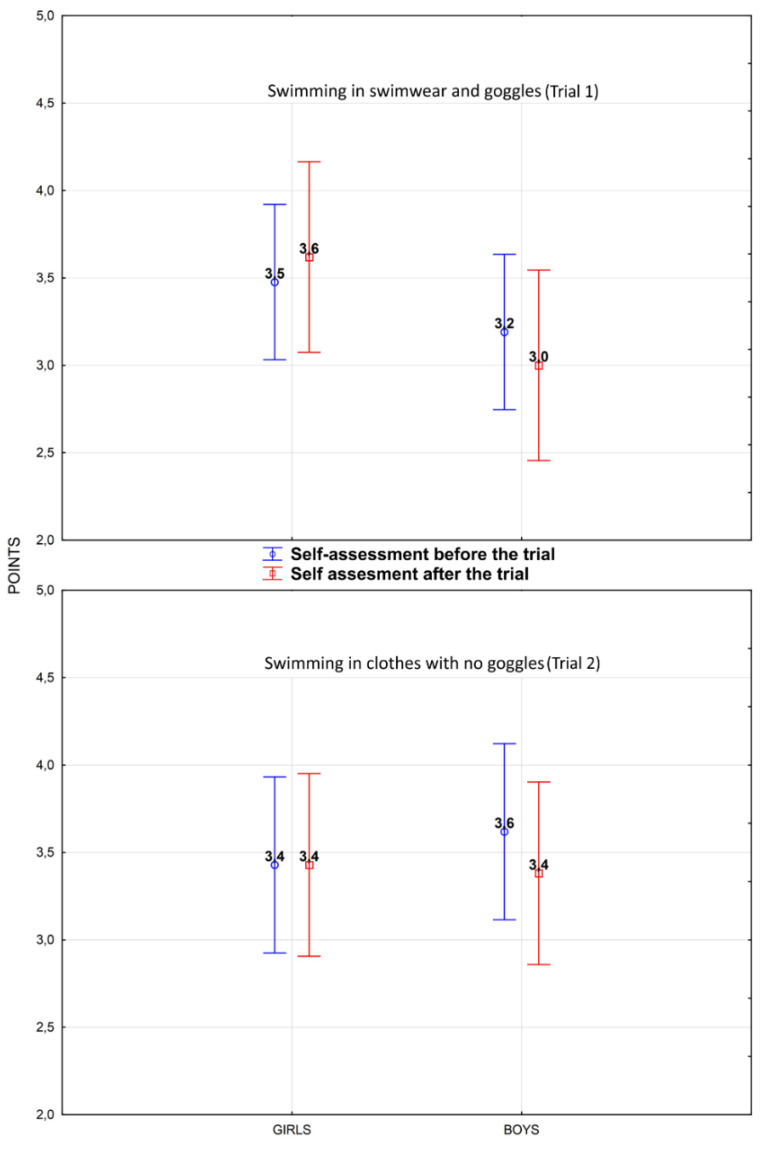
Comparison of mean self-assessment scores of girls and boys before and after swimming trials while wearing swimwear and goggles and while wearing plain clothes without goggles.

A comparison of self-assessment scores between swimming in swimwear and goggles and swimming in a more challenging situation wearing clothes and lacking goggles showed a statistically insignificant tendency that girls assessed themselves higher when they swam in a swimsuit than when they swam in plain clothes, whereas the self-assessment of boys was higher when they swam in clothes (Table 3 and Figure 2). These findings were statistically insignificant, which is similar to the results of comparing self-assessment scores before and after the two trials (Table 3 and Figure 2). Boys and girls scored themselves higher after the trial than before the trial when swimming in a swimsuit. In boys, this tendency was also seen when swimming wearing clothes. Girls did not change their self-assessment before and after the challenge trial where they swam in clothes.

When swimming in a standard swimsuit, the results of self-assessment before and after the trial were significantly (*p* < 0.01) correlated in both girls (R = 0.82) and boys (R = 0.79). For the more challenging trial (swimming in clothes without goggles), the correlations were also significant for both groups, but the correlation for boys (R = 0.91, *p* < 0.01) was more significant than that observed for girls (R = 0.6, *p* < 0.05). In general, in both groups and in both trials, the higher the self-assessment score of swimming skills before the trial, the higher the self-assessment of swimming skills after the trial, and vice versa. On the other hand, not all of the girls who took part in the experiment assessed themselves at the same level when swimming in clothes without goggles.

Girls assessed themselves higher than the experts in both swimming situations (in swimwear and goggles and clothes without goggles) as well as before and after both of these trials, but these differences were not statistically significant (Table 4). The self-assessment scores of boys were significantly lower than the scores given by experts for both trials before and after the trials. The boys’ self-assessment in the more challenging trial was more similar to the experts’ verdict than in the standard swimming trial. The intergroup analysis showed no statistical differences between girls and boys (Table 4).

The positive correlations presented in Table 5 can be interpreted as follows: the better the score in the reliable (expert) assessment of distance covered by adolescents, the higher the adolescents’ self-perceived (self-assessment) swimming skill, and vice versa. A significant proportional relationship was found between the experts’ scores of crawl swimming in clothes and self-assessment of these skills before and after the trials, but only in the case of girls. In boys, the reliable (expert) score for front crawl swimming skills while wearing swimwear was not significantly correlated with higher self-assessment scores before and after the trials. 0.3 < |R| ≤ 0.5, or strong, |R| > 0.5. 0.3 < |R| ≤ 0.5, or strong, |R| > 0.5.

## 4. Discussion

The front crawl swimming skills of adolescents, both girls and boys, was evaluated under standard swimming conditions, while wearing a swimsuit and goggles, and under a realistic risk scenario simulation in which they swam in clothes without goggles. The potential risk scenario was included to verify the protective value of these skills. The self-assessment scores provide insight into the adolescents’ subjective judgment of their own swimming skills’ level. The comparison of self-assessment scores before and after the trials enabled the evaluation of adolescents’ ability to learn from the experience gained from practical implementation of the task in order to accurately assess their swimming skills. A comparison of self-assessment scores representing the adolescents’ perceived swimming skill, with scores given by experts representing their real skills level, was also performed. This enabled an evaluation of the accuracy of adolescents’ self-assessment of their own swimming skills. Its accuracy, diagnosed during the experimental trials, was considered representative of their water competencies.

Comparison of the crawl swimming distance covered by adolescents in the current study (Figure 1 and Table 2) with results obtained by other studies (in which physical education students were able to swim for 25 m [16], children from primary school could swim for 27.5 m [20], and 10-year-olds could swim up to 20 m [15]) shows that our respondents demonstrated poorer crawl swimming skills. In the statistical analysis, the boys did not outperform the girls when swimming in swimwear or plain clothing in terms of distance (Figure 1). Nevertheless, it should be emphasised that a 5-metre difference could be enough to save a person in a life-threatening situation [4,8,11,12]. These results could be explained by the fact that, as teenagers, girls tend to be more biologically advanced than boys, while the onset of puberty in boys generally favours the development of gross motor skills. Moreover, considering social and cultural domains, boys tend to be encouraged to develop physical skills to a greater extent than girls, primarily through organised practice of psychomotor activities [35]. Furthermore, Burnett et al. [36] reported that the development of physical skills is influenced by the choice of preferred activities, which varies according to gender. However, Rokita et al. [37] reported that girls choose swimming as their preferred activity more often than boys.

The importance of swimming competencies while wearing plain clothing as a drowning prevention strategy has been emphasised in a number of studies [4,12,16]. In the current study, adding plain clothing did not significantly influence the swimming distance covered by boys or girls (Figure 1 and Table 2). It seems that the protective value of the participants’ swimming skills, verified in a potentially realistic risk scenario (swimming in clothes without goggles), could be assessed positively. However, when considering the short distance covered by the examined group, which indicates an overall low swimming skills level, this assessment seems unreasonable. These results appear to be consistent with the explanation proposed by Moran [16], who reported that even poor swimming skills may enable individuals to enact an emergency strategy, whereby their increased motivation could be translated into an enhanced ability to swim in unknown or unfamiliar conditions such as swimming in plain clothes. The adolescents in our study may have perceived increased resistance and put more effort into the clothed trial, which is similar to the subjects in the study by Moran [16], who swam wearing a flotation device. However, this has little in common with the real protective value of these skills in a life-threatening situation. 

No statistically significant differences were found in the self-assessment scores of adolescents’ swimming skills in both the standard and challenging swimming situations. The self-assessment scores did not differ before and after each of the previously mentioned trials (Table 3). However, statistically unverifiable self-assessment scores (Figure 2, Table 3 and Table 4) suggest that girls are more aware of their deficits when swimming wearing clothes, and, therefore, have a more reflective approach for assessing their swimming skills than boys. In boys, the higher self-judgment of swimming skills in clothes is likely the result of a spontaneous act of courage stimulated by the challenging nature of the task [3].

An accurate self-assessment of one’s own swimming skills level has recently been identified as an essential part of water competencies [4]. By initiating self-reflection [38], relevant self-assessment promotes a responsibility for action in and around water [39] in addition to reducing negative behaviours [40], which, thereby, represents a tool for drowning prevention. In regards to the analysis of the ability of adolescents to accurately assess their own swimming skills (Table 4), the girls’ assessment of perceived swimming skills was significantly closer to the scores given by experts. Boys, on the other hand, significantly underestimated their swimming skills (Table 4). To the contrary, in girls, no changes in self-assessment scores before and after the trials were found. Boys, however, seemed to gain experience (learn) directly during the practical implementation of the tasks, which resulted in a more accurate self-assessment after the trial (Table 4). 

This finding suggests that a more profound (evolved from practical experience) awareness of their own swimming skills could be an important step for developing water competencies. Nevertheless, the instinctive behaviour of girls could not be depreciated because it may reduce undesirable attitudes and behaviours, which contributes to increased water safety. The boys’ nature makes them more prone to risky behaviours, which means they may adopt undesirable attitudes and create dangerous situations in and around water [3]. More than twice as many males as females under the age of 25 years drown globally [1]. However, boys who participated in this study, fortunately, did not overestimate their real swimming skills (Table 3), especially considering that this is one of the greatest threats to safety [10,39].

Our results suggest that the studied group of adolescents learned how to swim, but did not acquire the knowledge and relevant experiences to be fully aware in terms of evaluating their own skills under different swimming conditions. Therefore, these adolescents would potentially belong to the group of more than 20% of children aged between 5 and 15 years who experience an emergency episode in water and are “able to swim” [1]. Swimming skills themselves are not sufficient to prevent drowning, especially when the level of these skills is poor. Therefore, water competencies, based on integration of affective, cognitive, and psychomotor competencies, are far more beneficial for safety in water [4]. 

Regarding the selected aspects of water competencies considered in this study, the group of adolescents would be considered to have poor water competencies. However, the results showing a higher score in the experts’ assessment of swimming skills go hand-in-hand with the adolescents’ self-assessment (Table 5) and provide an optimistic outlook on the water safety awareness of young people. According to Wiesner [2], it can be assumed that adolescents are mentally ready for education in the field of water competencies. Previously, these adolescents were likely only taught basic swimming skills. Consequently, they have not learned all aspects of water competencies, including the activation of critical thinking and the ability to improve their behaviours related to water. Moreover, they were not given the opportunity to develop metacognitive skills [38].

### Limitations of the Study

Firstly, the research group included 42 persons (21 girls and 21 boys) taking part in this study, which was not large, but was similar to the number of subjects researched in similar studies (i.e., 45 children [20], 37 and 40 students [11,16], or only six swimmers) [18]. In this context, knowing that limitation in the research group extent and the value of the statistical analysis applied, we are convinced that it did not depreciate the value of the results obtained. 

The research group was randomly selected from three schools. The low frequency of the lessons (thirteen 45-min lessons held once a week) has not promoted building up strong relationships between participants. There were no situations nor behaviors observed between the girls and the boys that could indicate any positive and negative emotions, which may have influenced the quality of tasks performed during the entire swimming course. Under such circumstances [41], the lack of separation of the boys and the girls into two research groups could not influence the reliability of the raw data acquisition. 

We are convinced that swimming 25 m is too short to prove the swimming skills with a reliable certification. However, in this study, we did not attribute the five points score (covering 25 m) obtained by participants as a score confirming their swimming abilities, skills, or water competences. The level of swimming skills of these adolescents was assessed in the same manner as in other prior studies [11,12,16]. Moreover, Brenner and Trumble [42] reported that lack of ability to swim a distance of 4.5 m is already a serious factor of drowning risk among children.

The choice of front crawl as a swimming technique was tested in this study. The adolescents took part in the short swimming course. Therefore, due to the time limitations, it had been decided to teach them only to swim backstroke and front crawl. It was mentioned before that front crawl is not an economical swimming stroke from the perspective of safety, but, on the other hand, it has higher utility value than backstroke because of better spatial orientation when swimming in open water. It can be assumed that front crawl is a more natural swimming stroke from the point of human motor abilities in comparison to breaststroke (due to alternating propulsive movements). These arguments provide the thesis that front crawl is the easiest form of frontal swimming to learn and to teach than breaststroke. In the context of swimming wearing clothes, this thesis is alternative to the suggestion that the breaststroke should be considered for learning very early in the aquatic education curriculum [20]. It possible that front crawl is more affected while swimming wearing only clothes because of the recovery of the “heavier” arm over the water [20]. Further research concerning the utility value of basic instead of a standard form of front crawl (e.g., “dog paddle”) and breaststroke (e.g., lack of dorsal feet flexion) should be considered in the future. This idea seems to be more intriguing due to the assumption that the skills in the various strokes (e.g., breaststroke, front crawl) mastered at the learning process are not necessarily linked with the ability of each student to adapt these skills to a challenging condition (swimming in clothing) [8].

## 5. Conclusions

The adolescents’ deficits in front crawl swimming skills likely made them unable to assess the real protective value of these skills in this study. Gender differences in the adolescent group were observed, specifically the accuracy of self-assessment of their swimming skills in both typical and challenging situations. The girls were likely aware of their deficits when swimming in clothes, and anticipated the dangerous consequences arising from these situations. Boys, who are usually more courageous than girls, underestimated their swimming skills in both situations. Nevertheless, they likely were more open-minded to learn from experience gained from practical implementation of the challenging task.

The results of this research did not provide compelling arguments to compare the level of water competencies of girls and boys in terms of water safety. It would appear that all adolescents are in need of more comprehensive aquatic education. They should be educated in total water competencies instead of merely in swimming skills. This research highlights some future direction of educational intervention aimed at improving water awareness of adolescents. For girls, enhanced “water readiness” is needed to broaden their abilities in adapting their swimming skills to nonstandard conditions, whereas aquatic education for boys should be focused on developing self-reflection and promoting the responsible use of acquired skills in and around water.

In this context, the need for educational institutions to properly educate young people in water competences needs to be emphasised. Swimming education of school-aged children and adolescents based on swimming skills themselves, but on the protective skills, self-confidence, self-rescue, and lifesaving competencies is of key importance in preventing drowning.

## Figures and Tables

**Table 1:** Assessment scale of crawl swimming skills and classification of self-assessment results obtained by the participants.

Variable for Swimming Skills Assessment		Evaluation Scale (Points)					
		0	1	2	3	4	5
Reliable expert assessment of distance covered (m)	Swimwear/goggles	0	5	10	15	20	25
	Clothes/no goggles						
Self-assessment of swimming in swimwear (points)	Before trial	0	1	2	3	4	5
	After trial						
Self-assessment of swimming in clothes (points)	Before trial	0	1	2	3	4	5
	After trial						

**Table 2:** Differences in the reliable (expert) assessment scores of swimming skills in girls and boys under standard (Trial 1 - wearing swimwear and goggles) and challenging (Trial 2 - wearing clothes without goggles) conditions.

Variable Tested	Student’s *t*-Test					
	Difference in Means	t	*p*	Difference in Means	t	*p*
		Girls			Boys	
Trial I, Swimwear and goggles	0.0952	1.4509	0.1623	−0.0000	−0.0000	1.0000
Trial II, Clothes without goggles						
	**Newman-Keuls Post Hoc Test** **Girls vs. Boys (*p* Value)**					
Trial I, Swimwear and goggles	0.0925					
Trial II, Clothes without goggles	0.0707					

Statistical significance was considered at *p* < 0.05.

**Table 3:** Differences in self-assessment scores of girls and boys before and after swimming tests under standard (Trial 1 - wearing swimwear and goggles) and challenging (Trial 2 - wearing clothes without goggles) conditions.

Variable Tested		Student’s *t*-Test					
		Difference in Means	t	*p*	Difference in Means	t	*p*
			Girls			Boys	
Trial I, Swimwear and goggles	Before the trial	−0.1429	−0.3838	0.7032	−0.1739	0.5345	0.5957
	After the trial						
Trial II, Clothes without goggles	Before the trial	0.0000	0.0000	1.0000	−0.3043	0.9526	0.3460
	After the trial						
Before the trial	Trial I, Swimwear and goggles	0.0476	0.1277	0.8990	0.4783	−1.6464	0.1068
	Trial II, Clothes without goggles						
After the trial	Trial I, Swimwear and goggles	0.1905	0.4802	0.6337	0.3478	−0.9895	0.3278
	Trial II, Clothes without goggles						
		**Newman-Keuls Post Hoc Test** **Girls vs. Boys (*p*-Value)**					
Trial I, Swimwear and goggles	Before the trial	0.0707					
	After the trial	0.1124					
Trial II, Clothes without goggles	Before the trial	0.5917					
	After the trial	0.8970					

Statistical significance was considered at *p* < 0.05.

**Table 4:** Differences in self-assessment scores of girls and boys before and after swimming tests under standard (Trial 1 - wearing swimwear and goggles) and challenging (Trial 2 - wearing clothes without goggles) conditions.

Variable Tested		Student’s *t*-Test					
		Difference in Means	t	*p*	Difference in Means	t	*p*
		Girls			Boys		
Trial I, Swimwear and goggles	Before the trial	−0.0476	−0.0889	0.9295	1.2174	3.3864	0.0015 ^2^
	After the trial	−0.1905	−0.3449	0.7319	1.3913	3.5656	0.0009 ^2^
Trial II, Clothes without goggles	Before the trial	−0.0952	−0.16873	0.8668	0.7391	2.0439	0.0470 ^1^
	After the trial	−0.0952	−0.16873	0.8668	1.0435	2.7222	0.0093 ^2^
Before the trial	Trial I, Swimwear and goggles	0.0000	0.0000	1.0000	0.7391	2.0439	0.0470 ^1^
	Trial II, Clothes without goggles	0.0000	0.0000	1.0000	1.0435	2.7222	0.0093 ^2^
After the trial	Trial I, Swimwear and goggles	−0.1429	−0.2609	0.7955	1.2174	3.3865	0.0015 ^2^
	Trial II, Clothes without goggles	−0.2857	−0.5066	0.6153	1.3913	3.5656	0.0009 ^2^
		**Newman-Keuls Post Hoc Test** **Girls vs. Boys (*p*-Value)**					
Trial I, Swimwear and goggles	Before the trial	0.3630					
	After the trial	0.1121					
Trial II, Clothes without goggles	Before the trial	0.5917					
	After the trial	0.8971					

^1^ Statistical significance was considered at *p* < 0.05. ^2^ Statistical significance was considered at *p* < 0.01.

**Table 5:** Pearson correlation coefficients between reliable (expert) assessment scores and self-assessment scores of girls and boys before and after swimming tests under standard (Trial 1 - wearing swimwear and goggles) and challenging (Trial 2 - swimming in plain clothes without goggles) conditions. Tables should be placed in the main text near the first time they are cited.

Experimental Trials		Girls	Boys
Trial I, Swimwear and goggles	Before the trial	0.5194 ^1^	0.4821 ^1^
	After the trial	0.5713 ^1^	0.5437 ^1^
Trial II, Clothes without goggles	Before the trial	0.4227 ^1^	0.3783
	After the trial	0.4899 ^1^	0.1975

^1^ Statistical significance was considered at *p* < 0.05.
